# Stakeholder engagement to ensure the sustainability of biobanks: a survey of potential users of biobank services

**DOI:** 10.1038/s41431-021-00905-x

**Published:** 2021-05-24

**Authors:** Corinna Klingler, Magdaléna von Jagwitz-Biegnitz, Ronny Baber, Karl-Friedrich Becker, Edgar Dahl, Cornelius Eibner, Jörg Fuchs, Maike K. Groenewold, Mara Lena Hartung, Michael Hummel, Roland Jahns, Romy Kirsten, Verena Kopfnagel, Regina Maushagen, Sara Yasemin Nussbeck, Anne Schoneberg, Theresa Winter, Cornelia Specht

**Affiliations:** 1 German Biobank Node, Charité Universitätsmedizin Berlin, Berlin, Germany; 2 Leipzig Medical Biobank, University Leipzig, Leipzig, Germany; 3 Institute of Laboratory Medicine, Clinical Chemistry and Molecular Diagnostics, University of Leipzig Medical Center, Leipzig, Germany; 4 Gewebebank des Klinikums rechts der Isar und der Technischen Universität München, Am Institut für Pathologie der TU München, Trogerstr. 18, 81675 München, Germany; 5 RWTH centralized Biomaterial Bank (RWTH cBMB), Institute of Pathology, RWTH Aachen University, Aachen, Germany; 6 Integrated Biobank Jena (IBBJ), Institute for Clinical Chemistry and Laboratory Diagnostics, University Hospital Jena, Am Klinikum 1, D-07747 Jena, Germany; 7 Interdisciplinary Bank of Biomaterials and Data Würzburg (ibdw), University Hospital of Würzburg, Straubmühlweg 2a, building A8/A9, 97078 Würzburg, Germany; 8 Research Unit of Molecular Epidemiology/Core Facility Biobank, Institute of Epidemiology, Helmholtz Zentrum München, German Research Center for Environmental Health, Neuherberg, Germany; 9 Central Biobank Charité (ZeBanC), Institute of Pathology, Charité Universitätsmedizin Berlin, Berlin, Germany; 10 NCT Liquid Biobank, National Center for Tumor Diseases and BioMaterialBank Heidelberg (BMBH), University Hospital Heidelberg, Heidelberg, Germany; 11 Hannover Unified Biobank, Hannover Medical School, Hannover, Germany; 12 Interdisciplinary Center for Biobanking-Lübeck (ICB-L), University of Lübeck, Lübeck, Germany; 13 Central Biobank UMG, University Medical Center Göttingen, Göttingen, Germany; 14 Integrated Research Biobank Greifswald, University Medicine Greifswald, Greifswald, Germany

**Keywords:** Social sciences, Ethics

## Abstract

Biobanks are important infrastructures facilitating biomedical research. After a decade of rolling out such infrastructures, a shift in attention to the sustainability of biobanks could be observed in recent years. In this regard, an increase in the as yet relatively low utilisation rates of biobanks has been formulated as a goal. Higher utilisation rates can only be achieved if the perspectives of potential users of biobanks—particularly researchers not yet collaborating with biobanks—are adequately considered. To better understand their perspectives, a survey was conducted at ten different research institutions in Germany hosting a centralised biobank. The survey targeted potential users of biobank services, i.e. researchers working with biosamples. It addressed the general demand for biosamples, strategies for biosample acquisition/storage and reasons for/against collaborating with biobanks. In total, 354 researchers filled out the survey. Most interestingly, only a minority of researchers (12%) acquired their biosamples via biobanks. Of the respondents not collaborating with biobanks on sample acquisition, around half were not aware of the (services of the) respective local biobank. Those who actively decided against acquiring biosamples via a biobank provided different reasons. Most commonly, respondents stated that the biosamples required were not available, the costs were too high and information about the available biosamples was not readily accessible. Biobanks can draw many lessons from the results of the survey. Particularly, external communication and outreach should be improved. Additionally, biobanks might have to reassess whether their particular collection strategies are adequately aligned with local researchers’ needs.

## Introduction

Biosamples and associated data are prerequisites for successful clinical and translational research. Where these are not available in the required quantity or quality or have not been collected in accordance with established ethical norms, this might delay research projects and subsequently important advances in (precision) medicine. Centralised biobanks working under well-defined and controlled conditions that ensure the adequate quality of biosamples and processes are therefore important infrastructures enabling and accelerating reliable biomedical research [1–3]. Recent genetic research has also been supported by biobank infrastructure and expertise [4–7]. In recent decades, the appreciation of the scientific value of human biobanks has led to a proliferation of such infrastructures [8, 9].

With institutions gaining more experience in operating human biobanks, one topic that has been discussed more prominently in recent years is that of sustainability—with a particular focus on an increase in utilisation rates [10–13]. In an international survey of 276 biobanks, more than half of the participating biobanks reported their utilisation rate to be 10% or lower [12]. It has been claimed that under-utilisation is not just a practical, but also an ethical issue. An argument can be made that human biobanks make an implicit promise to donors that their biosamples will be used in biomedical research and breaking this promise might undermine public trust [14, 15].

Various recommendations have therefore been formulated to boost the utilisation rates of biobanks. Among them count a change in potentially overly restrictive access policies, improving the communication of biobanks or building/joining a sample locator service [16]. However, these strategies are often formulated or even implemented without consulting the relevant stakeholders: potential users, i.e. researchers from academia or industry not yet using biobank services. Without understanding their perspectives, it might not always be clear which strategies will be most effective in increasing utilisation rates. The importance of adequately engaging with stakeholders for successful biobanking has been pointed out by various publications accordingly [9, 15, 17, 18].

So far, however, engagement with the group of potential users has been very limited. Studies have investigated the attitudes of researchers involved in biobank research towards relevant ethical issues (e.g. communication of results) to ensure acceptance of proposed concepts and processes [19–21]. Surveys have also been conducted on the biosample needs of researchers in the US and their hypothetical willingness to request/contribute biosamples from/to a supra-institutional biobanking infrastructure [22–24]. When this study was conceptualised no one had yet aimed to specify the reasons of potential biobank users for or against collaborating with a human biobank. Since then, a comprehensive study has been conducted in the UK [25] and focus groups in the Netherlands provided some first qualitative data [26].

Upon ascertaining this gap in knowledge, the German Biobank Node (GBN) decided to address this question in collaboration with its partners from the German Biobank Alliance (GBA). GBN was founded in 2013; it represents the German biobanking community in the pan-European network ‘Biobanking and Biomolecular Resources Research Infrastructure—European Research Infrastructure Consortium’ (BBMRI-ERIC) [27] and coordinates the GBA consisting of 20 biobank sites. Since its implementation, GBN has worked with its GBA partners to develop various services and products to support the biobanking community [28]. An overview of the products and services developed is available on our website: www.bbmri.de/service/?L=1 (last visited on 30 March 2021). GBN/GBA initiated a survey among potential users across various biobank sites serving to improve the understanding of their biosample needs, the different strategies chosen for biosample acquisition and storage and reasons for or against collaborating with centralised human biobanks.

## Materials and methods

No consolidated guidance on the reporting of survey research has been published [29], but we have taken different best practice models into account in the presentation of our findings [30, 31].

### Study design and survey development

In our understanding ‘potential users’ include researchers who already acquired and/or stored biosamples from/in a biobank in the past and might continue collaboration in the future as well as those who have not collaborated with biobanks at all yet. Both groups were targeted with the survey and a system of filtering questions served to tailor the questionnaire to the respective sub-group. Our survey focused on academic researchers, not on those from the industry. To avoid discouraging researchers unaware or even critical of biobanks from participating (as they might not have felt addressed by it), the survey did not focus on biobanks directly, but rather on the acquisition, storage and use of human biosamples more generally.

No hypothesis could reasonably be formulated due to a lack of previous studies. An exploratory web-based survey was designed accordingly. The questionnaire had to be developed from scratch as no established tools were available for the particular questions we were interested in. However, we built on a survey developed by our partner organisation in the UK (BBMRI.UK) [25]. We needed the survey to continuously adapt questions based on respondents’ previous answers so as to reflect the experience of individual participants. Hence, the survey length varied. After giving informed consent, participants were asked at least five and at most 19 questions. To decrease attrition rates all questions were voluntary—except filter questions—which prompted a different number of respondents for different questions. The survey was carried out in an anonymous manner and addressed the following aspects: (1) information on the researcher, (2) demand for human biosamples and (3) acquisition of human biosamples. For researchers who did not obtain biosamples from pre-existing collections, we additionally addressed (4) the storage of biosamples.

For implementation of the survey, we used LamaPoll software (www.lamapoll.de), which is popular in Germany and operates in accordance with national data protection laws. The survey was pre-tested using probing interviews [32] with researchers and clinician scientists. Their comments were analysed and used to improve the practical functionality and intelligibility of our survey. It has been published in German (the original survey language) and English online to allow for transparency. The survey can be accessed via the following links: Survey in German: https://lamapoll.de/Publication_Survey_Potential_Users_German/; Survey in English: https://lamapoll.de/Publication_Survey_Potential_Users_English/. In addition, we have provided an outline of the question logic as supplemental material.

### Data collection and participants

**Fig. 1:**
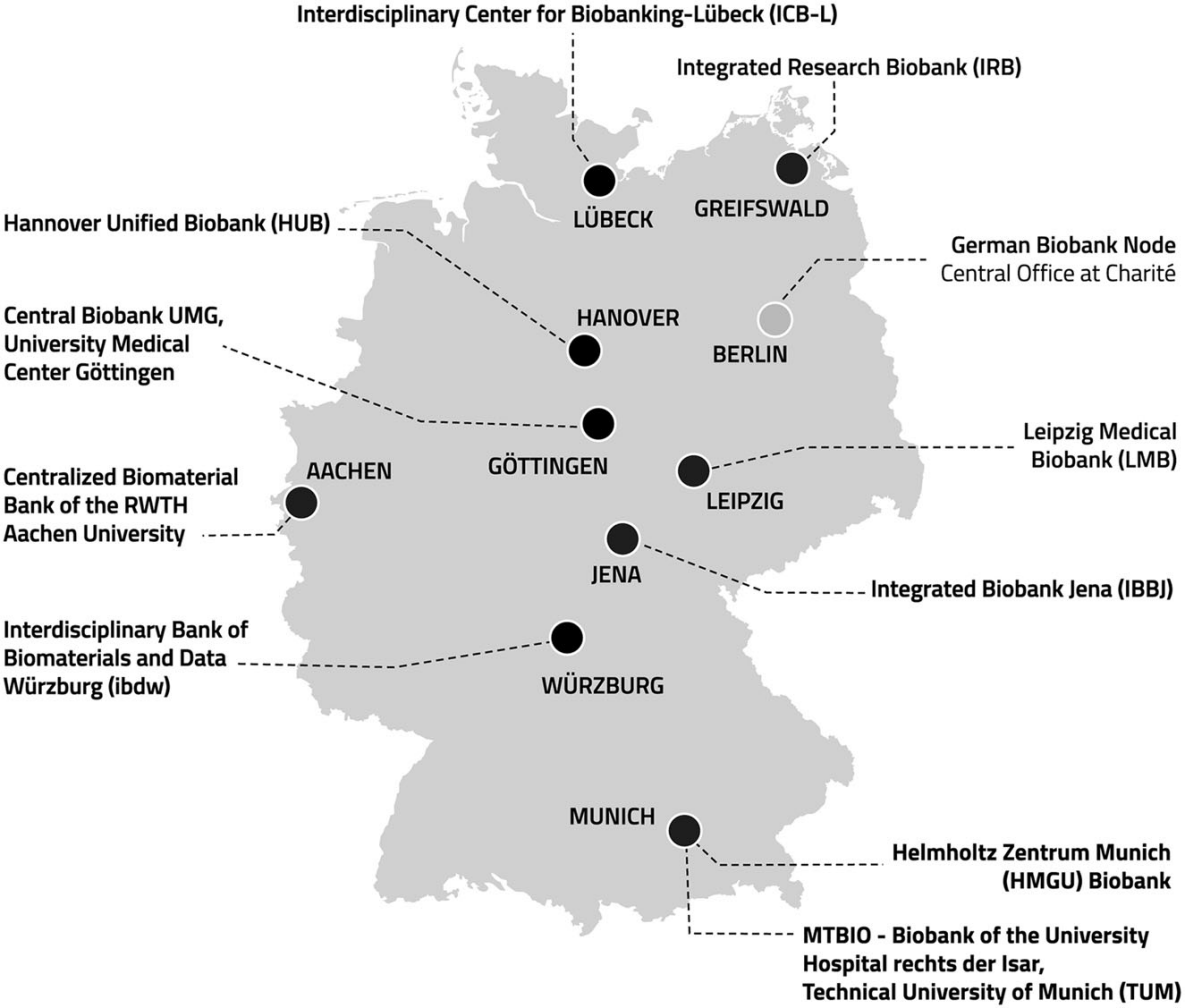
Institutions involved in either design (GBN) or implementation of the survey (GBA biobanks).

Ten biobanks of the German Biobank Alliance contributed data (see Fig. 1). They coordinated implementation of the survey at their respective institutions (university or research centre). As we had no mechanism to identify only those researchers working with human biosamples in advance, an invitation to participate in the survey was sent via email either to all researchers and clinician scientists at the respective institution *or* to the heads of departments and/or research institutions for further distribution among their research staff (snowball sampling). The choice of distribution mechanism was dependent on the local availability of relevant distribution lists, the willingness of the respective institutions to provide access to such lists and the available resources to compile such lists where they did not already exist. In one case, the university requested that heads of departments receiving the invitation were restricted to three access codes each that they could pass on to relevant staff. In the other nine cases, all interested researchers could access the survey. The survey was individualised for each site (i.e. the logo/name of the local biobank was added to the survey design).

The survey was sent out at different points in time between December 2018 and July 2019 depending on the local timeline. The survey was generally kept open for six weeks with a reminder sent after two weeks. Generally, a biobank employee (mostly the biobank director) signed the invitation email. In one instance, the dean of research of the medical faculty signed the mail to demonstrate her support for the project.

### Data analysis

In accordance with the exploratory approach, we only conducted descriptive statistical analyses providing an overview of responses. Twice we analysed the difference in mean between groups using non-parametric tests. The open-ended survey questions were analysed using qualitative content analysis by CK [33]. Data were analysed using Excel and SPSS (quantitative analysis) and Word (qualitative analysis).

No correction method was applied to the data set. For ethical and methodological reasons, only those participants who finished the survey, i.e. submitted their answers at the end, were included. Participants did not always answer all questions, but as long as the survey was finished, we did not exclude any participants for unanswered questions.

### Ethics and data protection

Our survey was approved by the ethics committee (application number: EA1/205/18) and the data protection division at the Charité Berlin. We furthermore approached the local regulatory bodies at each of the ten biobank sites. At some institutions, additional approval was required from the ethics committee, data protection office and/or staff council which was granted in all cases.

On the first page of the survey, potential participants were asked for their informed consent. On this page, we provided the most important information about the survey including the purpose, institutions involved, data collection in an anonymous manner (including a note that IP addresses would not be registered) and the right to withdraw from the survey at any time before submitting responses. More detailed information was provided in an accompanying PDF document. The respondents gave informed electronic consent by ticking a box indicating that they had read the relevant information and were willing to participate in the study. Only those who ticked the box were permitted to proceed to the survey.

## Results

In total, 354 researchers fully completed the survey. The response rate could not be calculated as snowball sampling was used.

Participants were asked for their position in the research organisation. They were given the opportunity to select more than one position (therefore the numbers do not add up to 100%). 147 participants (42%) were research associates in a research group, 75 (22%) said they were conducting their own research projects, 125 (36%) were leading their own research group and 54 (16%) identified as heads of departments, institutes and/or other full professors. 99 participants (29%) were conducting research alongside their routine clinical work. Of those who participated in the survey, only 256 (72%) had used human biosamples in their research projects in the last three years. Those not working with human biosamples provided the following reasons: ‘I do not need biosamples for my research’ (*n* = 39), ‘I use biosamples of animal origin’ (*n* = 29), ‘I plan to use human biosamples in future projects’ (*n* = 19), ‘Access to human biosamples involves too much effort for me’ (*n* = 7) and ‘Miscellaneous reasons’ (*n* = 4).

### Use of biosamples

**Fig. 2:**
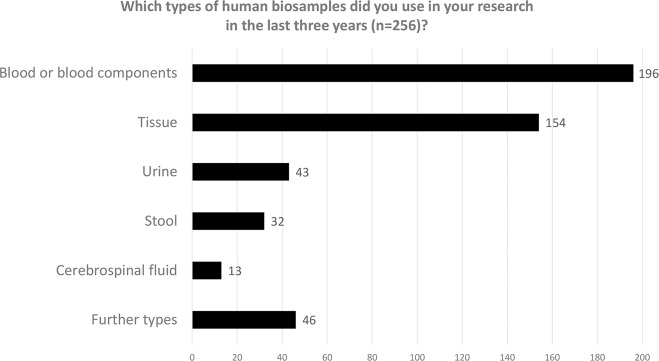
Biosamples used by participants in the last 3 years.

We asked researchers working with human biosamples about the types of biosamples they had used in the last three years (see Fig. 2). The majority used blood or blood components (*n* = 196) or tissue (*n* = 154) in their research projects. Fewer had worked with urine (*n* = 43), stool (*n* = 32), cerebrospinal fluid (*n* = 13) or other types of specimens (*n* = 46) which participants could specify by responding to an open question. An overview of the responses is provided in Table 1. In addition, we asked for the quantity of biosamples (aliquots) used in the last three years. 26 respondents (10%) had used nine or fewer biosamples, 53 participants (21%) had used 10–49 biosamples in their research, 36 (14%) had put 50–99 biosamples to use, 73 (29%) had worked on 100–499 biosamples and 67 (26%) had employed more than 500 biospecimens.

**Table 1:** Further biosamples processed by participants in the last three years (the numbers in brackets indicate how often participants mentioned the respective type of specimen).

From the oral system:
• Saliva (7)
• Teeth (3)
• Sulcular fluid (1)
• Dental biofilm (1)
From the respiratory system:
• Bronchoalveolar lavage/BAL (4)
• Nasal swab or lavage (3)
• Sputum (3)
• Nasal epithelial cells (1)
From the reproductive and lactation systems:
• Breast milk (3)
• Follicular fluid (1)
• Sperm (1)
• Cord blood (1)
From the optic system:
• Vitreous body (2)
• Various liquids of the eye (1)
From the locomotor system:
• Complete joints (1)
• Joint punctate (1)
Further:
• Bone marrow/bone marrow-derived stem cells (3)
• Ascites (1)
• Pancreas (1)
• Myocardium (1)
• Cerebral tissue (1)
• Mucin (1)
Further (unspecific):
• Swabs (5)
• Cells (4)
• DNA/RNA (2)
• Isolated bacteria (1)
• Muscle (1)
• Body parts (1)

### Acquisition of biosamples

In the next step, we wanted to know about the acquisition of biosamples. We first asked who was responsible for deciding about acquisition strategies. About half of the participants (*n* = 126, 50%) decided about acquisition strategies themselves, 27% (n = 68) of respondents indicated that those decisions were taken by the lead of the research group, in 14 % (*n* = 36) of the cases the head of the department, institute or chair took these decisions and in 9% (*n* = 24) it was decided by further stakeholders. Further stakeholders were stated (open question) to be collaboration partners (unspecified), collaborating biobanks, collaborating clinical staff, sponsors of studies, the study lead/PI or the study consortium.

**Fig. 3:**
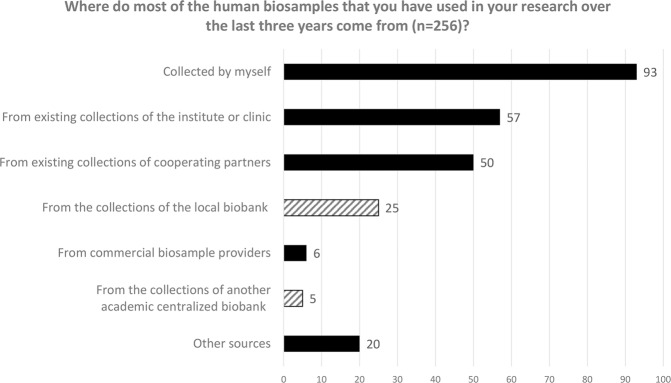
Acquisition strategies chosen by participants in the last 3 years. The striped pattern is used for answers where a biobank was the source.

We then asked who provided most of the human biosamples used in research (see Fig. 3). Only 12% (*n* = 30) of the biosamples were acquired via a centralised academic biobank, whereas 36% (*n* = 93) of the samples were collected by the researchers themselves, 22% (*n* = 57) came from existing collections of the institute or clinic and 20% (*n* = 50) were provided by collaborating partners. Very few researchers resorted to commercial providers of biosamples (2%, *n* = 6) or ‘other sources’ (8%, *n* = 20). Participants specifying these ‘sources’ (open question) most often mentioned clinical partners who collected biosamples for them, often as part of routine clinical care. They also referred to collaborating partners establishing new collections, voluntary donations and named specific institutions they had worked with. Participants were then asked to rate on a seven-point scale with only the highest and lowest rank defined (1 = ‘Not at all’ to 7 = ‘Entirely’) to what extent the acquisition strategy chosen had met their needs. While those who had obtained human biosamples from a biobank indicated that they were quite satisfied (*n* = 30, mean = 5.97, standard deviation/SD = 1.10), participants who had not acquired biosamples via a biobank were slightly less satisfied (*n* = 219, mean = 5.60, SD = 1.35). However, this difference in mean satisfaction is not significant (Mann–Whitney *U* Test, *p* = 0.202).

Those who had obtained their biosamples from a centralised biobank (*n* = 30) were asked why they had decided to acquire biosamples from the respective biobank (more than one option could be selected). The reasons most often given were the high quality of biosamples (*n* = 18), the only possibility to obtain relevant biosamples (*n* = 14), additional clinical data (*n* = 13) and pre-existing collaboration (*n* = 13). Those who had gained access to biosamples via other sources were asked about their awareness of the services of their institution’s centralised biobank. Interestingly, only 122 out of 226 respondents (54%) knew of the biobank and its services. We wanted to know from the 122 respondents aware of the biobank whether they had ever really considered obtaining biosamples from the local biobank and therefore actively engaged with its services and modalities. 66 respondents (54%) had actively considered acquiring biosamples from the respective biobank, the remaining 56 participants (46%) had not.

**Fig. 4:**
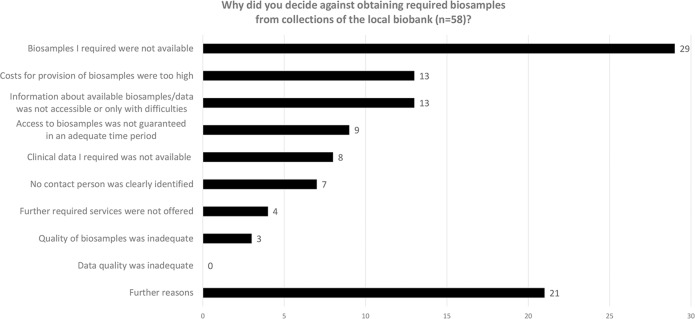
Reasons against obtaining required biosamples from a biobank. Only represents answers from those participants who actively considered collaboration with a biobank.

We asked the 66 participants who had considered working with the biobank on acquisition why they had decided against requesting biosamples from the biobank (see Fig. 4 for an overview). The reasons most often given were the limited availability of required (project-specific) biosamples (*n* = 29), the cost of provision (*n* = 13), the limited accessibility of information regarding available biosamples/data (*n* = 13) and that access to biosamples/data was taking too much time (*n* = 9). Further reasons could be specified in an open question. Participants found the processes of other partners easier/faster or more aligned with their individual needs and named research-related reasons (e.g. the need of fresh biosamples). Others stated that the required quantity of biosamples was either not available or already available via own collections. Some participants also pointed to the biobank or themselves still being new to the facility, referred to sceptical attitudes towards the local biobank or the difficulty of ensuring adequate funding.

**Fig. 5:**
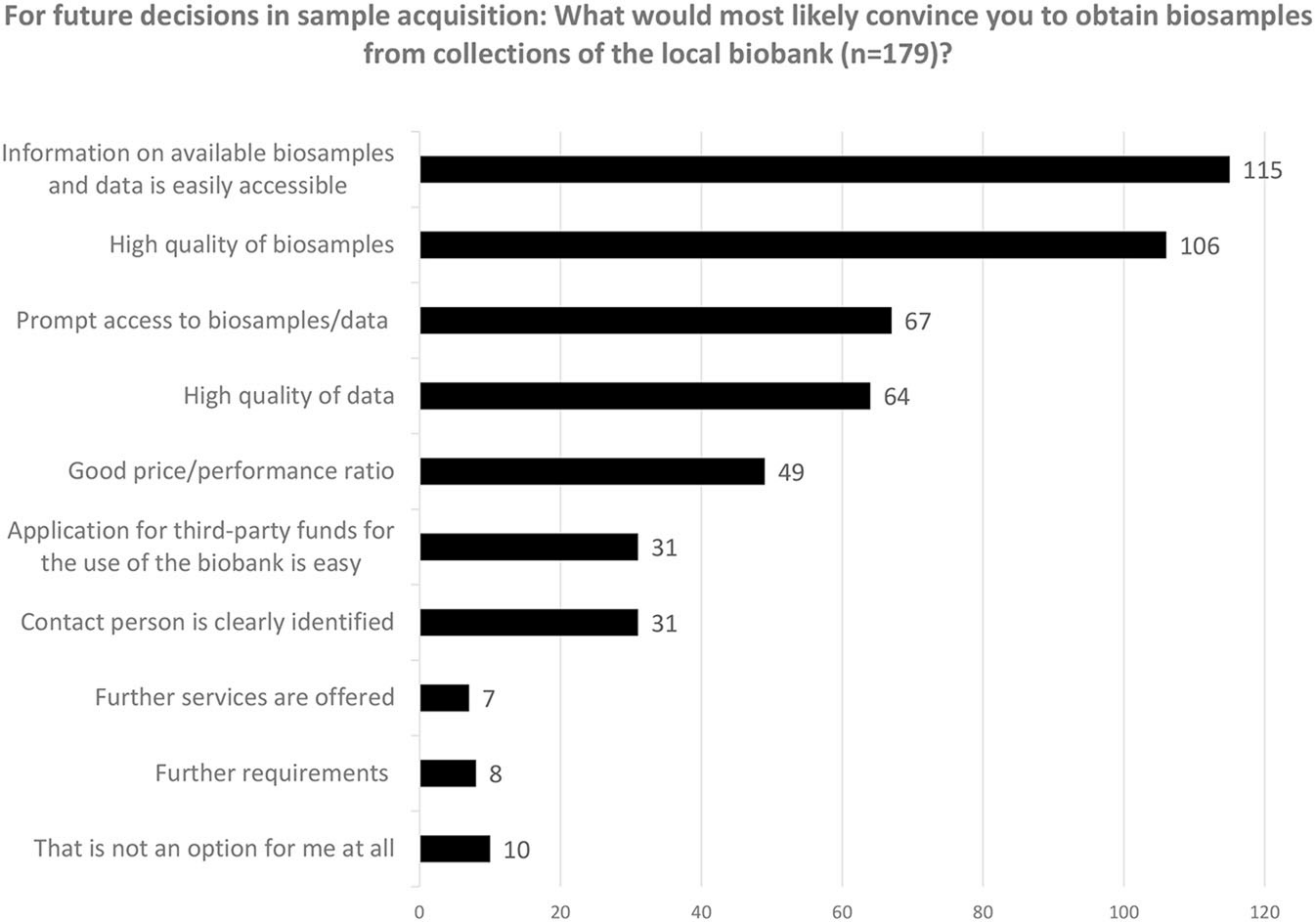
Decisive factors for collaborating with a biobank on future biosample acquisition. A maximum of three options could be selected per participant.

All 179 participants who were not (well) acquainted with the services of their institution’s centralised biobank - this group included those planning to work with biosamples in the future (*n* = 19), those who had conducted research with biosamples, but were not aware of the biobank at all (*n* = 104) and those who knew the biobank existed, but had not further engaged with their offers (*n* = 56) - were asked what would most likely convince them to request biosamples/data from the biobank in the future (see Fig. 5). The criteria most often selected (max. three per person) were the accessibility of information on biosamples/data (*n* = 115), high quality of biosamples (*n* = 106), prompt access to biosamples/data (*n* = 67) and high quality of data (*n* = 64). Participants who selected the option of ‘further requirements’ had the opportunity to further specify their needs (open question) and referred most often to (i) highly specific biosamples they needed (e.g. samples from vitreous bodies), (ii) adequate quantity of required biosamples or (iii) limiting the use of collected biosamples to their own research. Those who stated that collaboration with a biobank was no option at all held that conviction (open question) because their institution’s biobank—apparently—did not collect the right types of biosamples, respondents considered their own resources adequate, only needed fresh biosamples or anticipated additional complications and costs.

All participants—irrespective of their prior relationship with the biobank—could specify (open question) their interpretation of ‘prompt access’ if they had selected this criterion as important in choosing their biosample provider (*n* = 76). 56 of these 76 participants (74%) used the opportunity to specify and stated times ranging from one hour to six months as acceptable between the request for and delivery of biosamples (mean = 22.10 days; SD = 29.95 days - for the calculation, we assumed each month had 30 days). Participants were also given the opportunity to specify further services the biobank should offer in case they had indicated that this criterion was important which 12 participants did. Seven out of these 12 participants answered the open question and requested at least one of the following services: genomic analyses, preparation of tissue-sections for microscopic analyses, storage of teeth, cell isolation from freshly gained biosamples, serum centrifugation, utilisation of specific cryo-vials instead of straws and continuous collection and (one-by-one) delivery of single human biosamples (not just racks).

### Storage of biosamples

The 93 participants who indicated that they had collected the required biosamples themselves were asked to specify whether they had used their biosamples immediately (*n* = 58, 62%) or had to organise storage of the samples for later use (*n* = 35, 38%). The latter group was asked where they had stored their biosamples. The majority had used their own cooling units (*n* = 28, 80%), only four (11%) had stored their biosamples in a centralised biobank and three (9%) used cooling units of collaboration partners for storage. We asked the participants to what extent the chosen storage strategy had satisfied their needs (using the same scale as described above). Those using the service of a biobank were more satisfied (*n* = 4, mean = 6.5, SD = 0.58) than those who organised storage facilities themselves or with partners (*n* = 31, mean=5.35, SD = 1.72). The difference is not significant (Mann–Whitney *U* Test, *p* = 0.194).

The 31 participants who had not (yet) collaborated with the local centralised biobank were asked whether they were aware of the biobank and its services. Around 84% (*n* = 21/31) were not aware of the biobank’s service regarding quality-controlled storage of biosamples or were aware but had not actively considered collaboration with the biobank (*n* = 5/31). We asked them what would most likely convince them to collaborate with the local biobank in future storage decisions. The options (max. three) most often chosen were high quality and safety of the storage conditions (*n* = 16), the guarantee that collected biosamples will not be transferred to third parties without the collector’s permission (*n* = 14), biosamples can be accessed rapidly (*n* = 10) and a good price-performance ratio (*n* = 8).

### Further points raised

In a final open question, participants were asked whether they had further points they wanted to address regarding the acquisition, storage and/or use of (human) biosamples or regarding the survey itself. Twenty-seven participants provided feedback. Their comments fall in one of the categories identified in the following: Participants expressed interest in collaborating with the local biobank while voicing uncertainty whether the biobank could satisfy their specific needs (e.g. samples of skeletal muscle cells from the soleus muscle of patients and healthy people); collaboration with the biobank was not seen as promising for various reasons; the costs of storage were identified as a barrier; participants expressed a wish for a database to search for available biosamples/data (across institutions); participants claimed that the local biobank is not well known and should be more intensely publicised at the respective institution and further specific service requests (e.g. the biobank should perform informed consent procedures).

## Discussion

This is the first survey conducted outside the UK engaging with potential users of biobanks on this specific topic. Some of the findings should be taken into consideration in the future strategic decision-making of the biobanking community. First, we could show that only a low percentage of participating researchers obtained biosamples from an academic centralised biobank (around 12%). This finding should be alarming as it further calls into question the sustainability of biobanks. Consequently, it is important to develop strategies to increase collaboration between researchers and biobanks.

### Stakeholder engagement and outreach

Of the 226 respondents not collaborating with biobanks on acquisition, half of them were not aware of the existence of a centralised biobank at their institution. Furthermore, in open remarks, participants predominantly voiced their scepticism about whether the biobank can fulfil their specific biosample needs. A lack of required biosamples was also the reason most often given for not collaborating with the biobank as sample provider. Based on the survey we cannot say whether the lack was real or only perceived. A perceived lack could be addressed by improved communication (see below), however, it can be suspected that the collection strategies of many human biobanks (at least in Germany) might not always adequately consider the perspectives of the stakeholders they serve. Involving stakeholders—e.g. heads of relevant departments or principal investigators of clinical trials/research consortia—in decisions regarding current and future collection strategies might increase the awareness of biobank services and additionally ensure the alignment of strategic biobank decisions with local researcher needs. Biobanks have to understand that stakeholder engagement will be a prerequisite for sustainable management of biobanks.

These findings also emphasise the need for increased outreach activities to ensure researchers are aware of the biobank offers (e.g. presentation of the biobank and its services at meetings of relevant departments – for further suggestions, see reference [34]). These kinds of activities have to be implemented by biobanks locally and might have been neglected so far. Consequently, GBN has initiated a programme in order to support the biobanks of the GBA and beyond more actively in this matter: a short film presenting the rationale for (centralised) biobanks has been produced and workshops have been (and will be) organised to facilitate a regular exchange regarding successful outreach activities. The outcomes of the first outreach-workshop are being condensed into best practice guidelines and will be fed back to the biobanks as templates. GBN also facilitates the presentation of success stories highlighting the beneficial effects of collaboration between human biobanks and medical researchers on biobank websites and at various conferences (e.g. at the German Cancer Congress 2020) to increase awareness of high-quality human biobanking. However, these communication efforts are of no value, if offers are not aligned with stakeholder needs (see above). In addition, it would be desirable to evaluate the effectiveness of these and possibly further outreach activities which has to our knowledge not been done.

Whether limited awareness and lack of needed samples is also an issue outside of the German context is unclear as it has to our knowledge not been addressed. A study conducted in the UK [25] emphasised that researchers prefer local and/or known sample sources they can trust – which stresses again the importance and potential of engaging with the local stakeholder community. BBMRI-ERIC has engaged in a Europe-wide survey of researchers which will provide additional insights in this regard and indications where BBMRI-ERIC can actively support the European biobank community in the future - for more information see: https://www.bbmri-eric.eu/news-events/characterising-researchers-who-use-human-samples/ (last visited on 30 March 2021).

### Accessibility of information and costs

Participants not well acquainted with the local biobank emphasised the importance of the accessibility of relevant information (see Fig. 5). GBA has been developing the Sample Locator which allows searches for biosamples and data across participating institutions. It can be accessed here: https://samplelocator.bbmri.de (last visited on 30 March 2021). The development was supported by a usability study involving potential users of the search tool [35]. Once finalised, this database will significantly reduce the work for researchers of identifying and negotiating with potential biobank partners. In addition, the BBMRI-ERIC Directory which lists European biobanks and their collections can be a helpful resource [36]. It can be accessed here: https://directory.bbmri-eric.eu/ (last visited on 30 March 2021). However, the biobanks can also play a part in improving access to relevant information (e.g. contact details, access policies, specificities of informed consent documents) by updating their websites, which often contain only rudimentary information about the biobank’s processes (e.g. information regarding access policies is often limited [37]). Websites have been critically evaluated during the above-mentioned workshop and GBN is preparing templates to help biobanks improve their online communication.

Some participants also criticised the (supposed) costs of collaborating with centralised biobanks. The difficulties in ensuring long-term funding for the storage of biosamples and related data was mentioned, but also that costs for the (storage of) biosamples were considered too high. One participant even described prices as ‘absolutely outrageous’ in an open comment. In this regard, biobanks might have to communicate more clearly that quality-controlled secure storage—and accordingly also biosample quality—comes at a cost, which is beyond dispute. It might also be helpful if biobanks were transparent regarding their underlying cost models. One option might be to make cost tables and cost models available on their websites so that interested researchers can judge the soundness of calculations. GBN/GBA is working on a uniform cost model that can be used by its members—also to facilitate the calculation of long-term storage costs for researchers and funding agencies. Biobanks might also try to secure institutional funding to ensure costs are acceptable to the individual researcher. However, if stakeholders and/or the biobanks’ institutions do not judge the value they receive worth their money, this is a problem for the biobank in terms of financial sustainability [18]. This again emphasises the need for stakeholder engagement and ensuring services are tailored to the needs of researchers.

### Further points

Reassuringly, participants did not see the quality of biosamples provided by centralised biobanks as a problem. Only three of the 58 participants who actively engaged with biobank services stated that the quality of biosamples was a reason for them not to request biosamples from the local biobank (see Fig. 4). GBN/GBA have invested a great deal in recent years in order to improve the quality of biobanks with a quality management manual, regular round robin tests and friendly audits. These activities have been successfully implemented and positively received among the community, but these efforts should also be actively communicated, as biosample quality is one of the most important criteria for decisions in sample acquisition (see Fig. 5).

Lastly, the role of biobanks in the research community has been debated: [38] should biobanks focus on providing/storing biosamples or should they aim to become technological platforms offering additional services (e.g. tissue staining, genomic analyses)? In this regard, it is interesting that only a few participants have seen ‘further services’ as an important criterion for collaborating with a biobank. Even participants finding further services important often requested traditional core functions of a biobank to be adapted (e.g. use of specific storage tools) and not additional services to be offered. This should be considered by biobanks when developing future strategies. However, what is needed might vary locally and strategic planning should be aligned with the needs of the (local) stakeholder community.

## Limitations

Due to the exploratory nature of the survey, the representativeness of our findings cannot be clearly established. The survey was conducted at only ten research institutions. Whether researchers at other institutions have made similar experiences is unclear. Biobanks at other institutions might be even less well known as GBN member institutions have often a more advanced biobanking infrastructure as is average. Furthermore, researchers from the industry context have not been represented among the sample. Particularly questions regarding biosample storage that were only answered by a few participants should be handled with care. For reasons of transparency, the local biobank had to be named in the invitation email. It might be the case accordingly that those without any former engagement with biobanks were discouraged from participating in the survey as they might have assumed they cannot contribute anything. However, we tried to minimise this risk by focusing the text on biosample use for research purposes. The number of researchers working vs. not working with biosamples should definitely not be seen as representative. The invitation email for the survey clearly targeted researchers working with biosamples and it is therefore to be expected that these researchers were overrepresented. The question was necessary, however, to filter the stakeholder group we were interested in, as we could not be more specific in our recruitment.

Four biobanks could only access lists of high-level decision-makers (e.g. heads of departments, institutes and full professors) for distributing the survey invitation and had to rely on them to pass the email on to their subordinates. This might have led to an overrepresentation of high-level decision-makers among our sample. The structure of the survey along different functions of the biobank (acquisition vs. storage) might not have been intuitive for all participants and might have led to misunderstandings, although probing interviews showed the survey to be comprehensible.

## Concluding remarks

This exploratory study provides a preliminary overview of attitudes and perspectives of potential users on collaborating with biobanks. Biobanks should use these insights to tailor their services/activities to this important stakeholder group thereby ensuring sustainability.

## Supplementary information

Question logic of survey
