# Supplementary figures and images for: Stakeholder engagement to ensure the sustainability of biobanks: a survey of potential users of biobank services
Source: Eur J Hum Genet. 2021 May 24;30(12):1344–54. doi: 10.1038/s41431-021-00905-x (PMC9712417; doi:10.1038/s41431-021-00905-x)

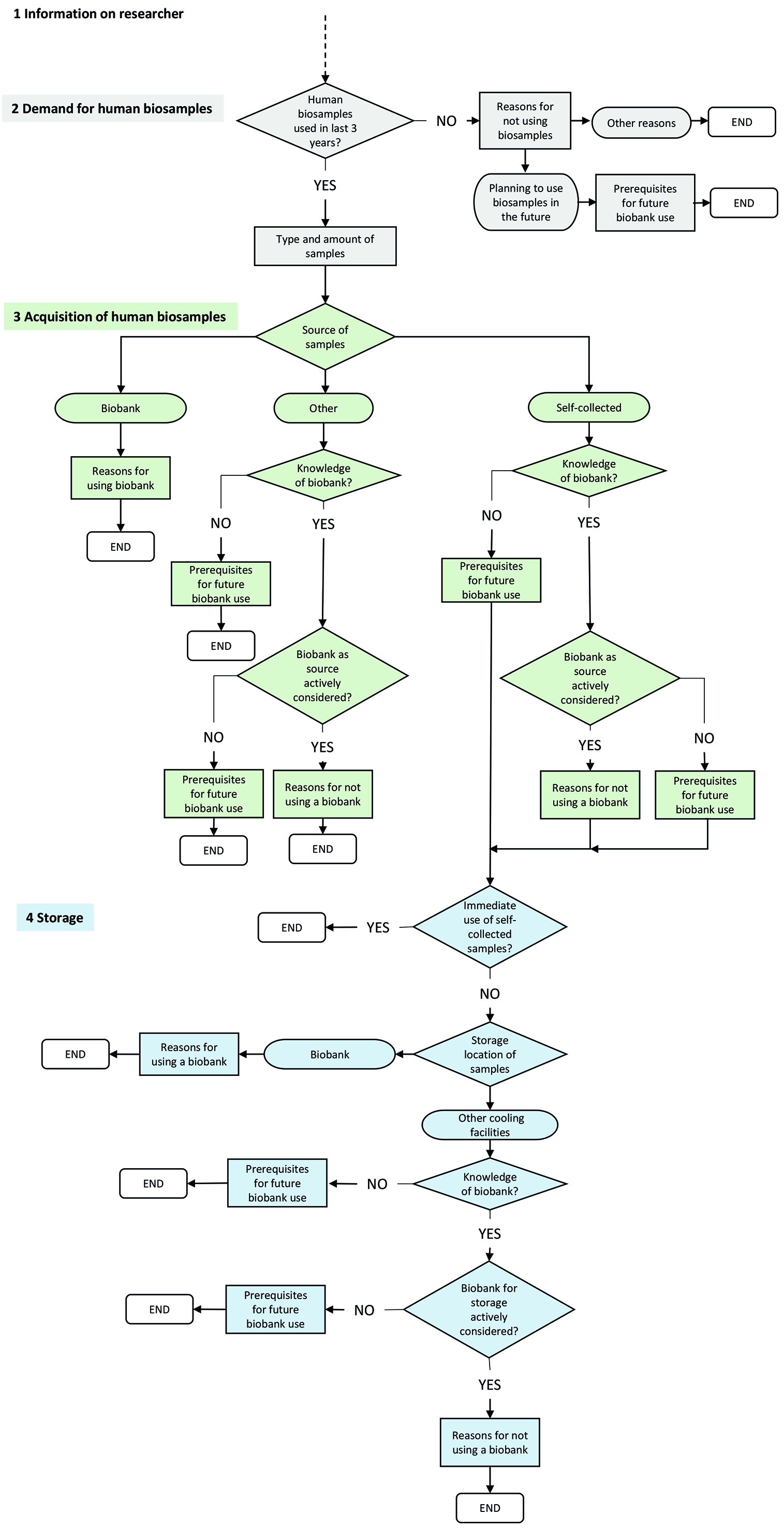

Supplement: Supplementary file 1 — Question logic of survey [file 41431_2021_905_MOESM1_ESM.tif]
